# The smoke detector principle: Signal detection and optimal defense regulation

**DOI:** 10.1093/emph/eoy034

**Published:** 2018-12-04

**Authors:** Randolph M Nesse

**Affiliations:** Center for Evolution and Medicine, Arizona State University, PO Box 871701, Tempe, AZ 85287-1701, USA

## DEFINITION AND BACKGROUND

The Smoke Detector Principle (SDP) explains why evolved systems that regulate protective responses often give rise to false alarms and apparently excessive responses.^1–3^ It gets its name because false alarms from the body’s protective systems are like smoke detector alarms—frequent minor annoyances that are necessary to avoid possible catastrophes. Anxiety, inflammation, pain, vomiting, anxiety, cough and diarrhea are protective responses whose costs tend to be small relative to possible catastrophic costs if no response is expressed when a danger is present, so false alarms or excessive responses are expected from optimal regulation systems. Error management theory uses a related argument to explain apparent errors in human cognition.^4^

Signal detection theory can specify the decision strategy that maximizes benefits in the face of uncertainty.^5^ The SDP describes the special case when the costs of a response are low, the costs of failing to express a needed response are large, and the presence of danger is uncertain. As the amount of information is increased, as reflected by a wider separation of noise and signal distributions, the percentage of false alarms can be reduced without compromising safety.

## EXAMPLES IN CLINICAL MEDICINE AND PUBLIC HEALTH

Panic attacks are emergency responses in the absence of danger. The decision to flee from a noise that could have been made by a lion depends on the cost of a response (CR), the cost of not fleeing if the danger is present (CD) and the probability that the noise was made by a lion (pD). If a response provides complete protection, fitness is maximized by expressing a response whenever pD> CR/CD.^3^ For example, a response that eliminates a 10 000-calorie cost of a lion attack will be worth its 100-calorie cost whenever pD > 1/100, i.e. whenever the noise indicates a 1% or greater chance that a lion is present. This means that 99% of responses will be false alarms that are nonetheless normal (Table 1).

**Table 1.** Decision making under uncertainty for a response that provides complete protection

	**Danger present**	**No danger present**
**Response**	**Hit** (correct response) Cost=CR	**False alarm** (incorrect response) Cost=CR

**No response**	**Miss** (no response) Cost=CD	**No response** (correct) Cost=None

The SDP can help guide clinical decisions about the use of medications to block responses such as anxiety, cough, vomiting, and fever. Such graded responses will often be more intense than is needed for the current situation; this helps to explain why it is often possible to use medications to relieve suffering safely. However, it calls attention to the drastic costs that occasionally result from blocking normal responses, for instance, pneumonia from excessive cough suppression after surgery. The SDP offers a useful framework for personalized medicine decision making.

**Conflict of interest**: None declared.
